# Genetic Association with Subgingival Bacterial Colonization in Chronic Periodontitis

**DOI:** 10.3390/genes9060271

**Published:** 2018-05-23

**Authors:** Franco Cavalla, Claudia Cristina Biguetti, Jessica Lima Melchiades, Andre Pantenuci Tabanez, Michelle de Campos Soriani Azevedo, Ana Paula Favaro Trombone, Marcelo Faveri, Magda Feres, Gustavo Pompermaier Garlet

**Affiliations:** 1 Conservative Dentistry Department, School of Dentistry University of Chile, Santiago 8380544, Chile; icavalla@odontologia.uchile.cl; 2 Department of Biological Sciences, Bauru School of Dentistry, University of São Paulo, Bauru 17012-901, São Paulo, Brazil; biguetti@usp.br (C.C.B.); jessicalimamel1903@gmail.com (J.L.M.); andrepetenucci@hotmail.com (A.P.T.); michelle_soriani@hotmail.com (M.d.C.S.A.); 3 Department of Biological and Allied Health Sciences, Sacred Heart University, Bauru 17011-160, São Paulo, Brazil; tromboneap@yahoo.com.br; 4 Dental Research Division, Department of Periodontology, Guarulhos University, Guarulhos 07115-280, São Paulo, Brazil; mfaveri@prof.ung.br (M.Fa.); mferes@ung.br (M.Fe.)

**Keywords:** chronic periodontitis, polymorphism, dysbiosis, biofilm

## Abstract

Chronic periodontitis is the most prevalent form of inflammatory destructive bone disease and has been affecting humans since antiquity. Evidence suggest that genetic factors can highly influence periodontitis risk, modulating disease elements such as the susceptibility to microbial colonization and the nature of subsequent host-microbe interaction. Several single-nucleotide polymorphisms (SNPs) have been associated with the occurrence of periodontitis, but the full range of genetic influence in periodontitis outcomes remains to be determined. In this context, this study comprises an analysis of possible correlation between periodontitis-related genetic variants with changes in the subgingival microbiological pattern performed in a Brazilian population (*n* = 167, comprising 76 chronic periodontitis patients and 91 healthy subjects). For the genetic characterization, 19 candidate SNPs were selected based on the top hits of previous large genome wide association studies (GWAS), while the subgingival microbiota was characterized for the presence and relative quantity of 40 bacterial species by DNA-DNA checkerboard. The case/control association test did not demonstrate a significant effect of the target SNPs with the disease phenotype. The polymorphism rs2521634 proved significantly associated with *Tannerella forsythia*, *Actinomyces gerencseriae*, *Fusobacterium periodonticum*, and *Prevotella nigrescens*; rs10010758 and rs6667202 were associated with increased counts of *Porphyromonas gingivalis*; and rs10043775 proved significantly associated with decreased counts of *Prevotella intermedia*. In conclusion, we present strong evidence supporting a direct connection between the host’s genetic profile, specifically rs2521634, rs10010758, rs6667202, and rs10043775 polymorphisms, and the occurrence of chronic periodontitis-associated bacteria.

## 1. Introduction

Chronic periodontitis is an infectious inflammatory disease characterized by progressive and irreversible damage to tooth-supporting structures. The initiating stimulus for chronic periodontitis is the tooth-attached subgingival biofilm that causes the activation of the host’s immune response [1,2,3]. While the host’s inflammatory immune response provides protection against the infecting agents’ dissemination, host mediators also stimulate local proteolytic and bone resorptive activities, which leads to periodontal tissue destruction [1,4,5,6]. Periodontitis is a complex disease, with etiologic factors acting at numerous levels: at the microbial level, based on the presence of dysbiotic microbial communities; at the host level, based on host response variations and genetic factors that may predispose to or protect from disease; and at the environmental level, factors that modify the host response in either a protective or destructive outcome [7,8,9].

Nevertheless, periodontitis is considered an infectious disease and the causative factor is the subgingival biofilm [10]. The subgingival biofilm is a complex community of several bacterial species that establish intricate ecologic relationships, organized in recognizable mutually supportive communities or complexes [11]. Recent analyses indicate that periodontitis is associated with a shift in the normal commensal oral microbiome towards distinct, but stable, dysbiotic communities associated with disease [12]. Thus, while some keystone microbes seem to trigger dysbiosis, disease results not from individual pathogens but rather from dysbiotic polymicrobial synergy, which disrupts the host-microbe homeostasis [10,13,14].

Indeed, even though periodontitis is regarded as an infectious disease, the extent and nature of the host inflammatory/immune response against the subgingival biofilm are the main determinants of disease outcome [4,15]. In this context, when the response is biased towards a T helper 1 cell (T_h_1), T helper 17 cell (T_h_17) or pro-inflammatory phenotypes, the effector mechanisms of the immune system tamper with the homeostatic balance of periodontal tissues, leading to a progressive destruction of tooth-supporting structures. Conversely, when the response is biased towards a regulatory phenotype, the tissue metabolism remains unhampered and the periodontium is preserved [1,16]. It is worth noting that the nature of the immune response is at least partially under genetic control [17,18]. While microbial and environmental factors characteristically modulate host responses in chronic periodontitis, evidence suggest that as much as 50% of the risk of suffering periodontitis can be determined by genetic factors [19,20] and that numerous disease-modifying genes may be involved in the pathogenesis of periodontitis by modulating the host’s response to infection [15,21].

Over the past decades, most of genetic association studies for periodontitis susceptibility have been based in the strategy of ‘candidate genes’, selected based on their theoretical involvement in key steps of the immune response or periodontal tissue metabolism [18,22]. However, usually after the identification of a ‘candidate single-nucleotide polymorphism (SNP)’, subsequent replication studies have revealed that the association is not necessarily consistent in populations with different ethnicities, which can be also impacted by variations in the study design (such as the disease definitions as well the nature of control subjects) [23,24,25]. Therefore, while few SNPs have been repeatedly associated with periodontitis in some populations [25], the full range of genetic influence in periodontitis outcomes remains to be determined. A new strategy based on the results of large genome wide association studies (GWAS) [26,27,28,29] overcomes some limitation of the classic studies by offering the crucial advantage of hypothesis-free gene selection criteria.

However, chronic periodontitis-focused GWAS relying exclusively on clinical criteria have had modest success in identifying risk genetic markers [30,31]. One possible cause for this difficulty is that most published GWAS in periodontitis have used sample populations presenting known confounding risk factors, such as smoking and diabetes, which lowers the statistical power of the analysis [32,33]. Also, even in studies that provide significant links between genetic variants and periodontitis outcomes, the association between SNPs and individual clinical readouts is modest, reinforcing the complex nature of periodontitis [34,35,36,37,38].

Consequently, the inclusion of secondary/surrogate microbiological outcomes in a recent GWAS has proven a more successful strategy, since changes in the subgingival biofilm structure could be considered proxy outcomes of the disease phenotype and offer a more sensitive screening strategy [33]. Despite limited microbiologic screening (which included a set of periodontal bacteria), the rationale behind the use of proxy outcomes to improve the odds of the identification of genetic risk determinants of periodontitis seems promising. Indeed, a series of studies describe the association of specific periodontal microbes with genetic variants [9,39,40,41,42]. However, a recent systematic review states that despite the increasing description that host genetic variants can affect the colonization by specific microbes, at this time there is no evidence that genetic polymorphisms are definitely associated with subgingival microbiota, reinforcing the requirement of additional studies focused in periodontal infectogenomics [43].

The use of microbiological surrogates in association with genetic data also seems suitable in the evolutionary context. Confounding risk factors such as smoking and diabetes have a major impact in chronic periodontitis risk and phenotype [44,45], yet such factors can be considered a ‘modern’ event and consequently would not comprise a significant evolutionary pressure towards the selection of periodontitis resistant/susceptible genotypes. On the other hand, the microbial biofilm is recognized as periodontitis’ primary etiologic factor and is regarded as the cause of periodontal disease in archaeological specimens [46,47], suggesting that microbial factors could in theory present some evolutionary impact in periodontitis resistance/susceptibility. Therefore, it is possible to hypothesize that genetic variations that facilitate colonization of subgingival biofilm by keystone microbes and/or facilitate the establishment of dysbiotic subgingival communities could act as primary risk factors for the development of periodontitis, since this initial event would subsequently lead to unbalance host-microbe homeostasis and afterward to tissue breakdown [7,48].

Thus, in this study, 19 candidate SNPs, selected based on the top hits of previous GWAS that included clinical and microbiological outcomes [27,28,33], were tested regarding their association with the occurrence of chronic periodontitis in a population free of major disease co-factors (smoking, diabetes, and other metabolic diseases) and with the presence and counts of 40 subgingival bacteria belonging to the classical subgingival pathogenic and commensal microbiological complexes [11].

## 2. Material and Methods

### 2.1. Sample Population

The chronic periodontitis sample (*n* = 76) was recruited in São Paulo state, southeastern Brazil, from patients referred to the Periodontal Clinic University of Guarulhos (UnG). Patients were examined by one experienced periodontist and scored for bleeding on probing (BOP), probing depth (PD) and clinical attachment loss (CAL). The chronic periodontitis diagnosis was based on the current classification of the American Academy of Periodontology [49]. The inclusion criteria were as follows: ≥ 30 years of age and a minimum of six teeth, with at least one site each with PD and clinical attachment level (CAL) ≥ 5 mm, as well as at least 30% of the sites with PD and CAL ≥ 4 mm and BOP [50]. A single-calibrated examiner performed all clinical evaluations and sample collection, following previously established methods and parameters [51]. Intra-examiner reliability was evaluated by Cohen’s kappa, by the repeated measures strategy. The examiner attained an almost perfect reliability (κ = 0.89) [52]. Age/gender matched healthy controls (*n* = 91, subjects presenting healthy gingival tissues, i.e., BOP < 10%; Gingival index (GI) < 1, no sites with PD > 3 mm or CAL > 3 mm), and were scheduled to restorative dentistry procedures [24] and recruited at the School of Dentistry University of Guarulhos and at the Bauru School of Dentistry University of Sao Paulo. Enrolled subjects provided informed consent that was approved by the Institutional Review Board. Subjects were excluded from the study if they were tobacco smokers (including former smokers), had medical history indicating evidence of known systemic modifiers of periodontal disease, were pregnant or nursing, were under or required treatment with antibiotics or anti-inflammatory drugs, and/or had received periodontal therapy in the previous two years. Clinical and demographical information of the sample population is summarized in Table 1. The classic ancestry stratification based on phenotypic features (such as skin pigmentation, hair color and texture, and the shape of the nose and lips) was not performed in this study due to the high individual ancestral variability observed in Brazilian population, which reflects a singular proportion of Amerindian, European and African ancestries in this mosaic genome and it is a poor predictor of genomic ancestry as estimated by molecular markers [53,54,55]. Despite the great diversity of the Brazilian population, the study population (both cases and controls) were recruited in the southeastern Brazil, where genomic ancestry has been found to be relatively uniform, and to be in relative uniformity with other geographical regions of Brazil [51]. Since no specific recruitment strategy based on ethnicity was adopted, we do not expect a biased genetic origin between the cases and controls groups.

### 2.2. Genotyping

Saliva was collected from all the participants at the enrollment session using a DNA Oragene OG-500 kit (DNA Genotek, Ottawa, ON, Canada), following the manufacturer’s instructions. DNA was extracted from participants’ saliva using QIAamp DNA Mini Kit (Qiagen, Hilden, Germany), according to the manufacturer’s guidelines. A spectrophotometer (Nanodrop 1000, Thermo Scientific, Waltham, MA, USA) was used to quantify and qualify the DNA samples. All isolated DNA samples were between 1.7 and 1.9 (260/280 nm ratio) and 1.9 and 2.1 (260/230 nm ratio). Genotyping was performed using TaqMan SNP genotyping assay (Applied Biosystems, Foster City, CA, USA), containing a 20× mix of unlabeled PCR forward and reverse primers as well as a VIC- and FAM-labeled allele discrimination probes. In this study, 19 SNPs (Table 2) were assayed and selected based on the top hits of previous GWAS that included clinical and microbiological outcomes [27,28,33].

Quantitative polymerase chain reaction (qPCR) was carried out in a 5 µL reaction mixture with 4 ng of genomic DNA and 2.5 µL of the Taqman genotyping PCR master mix (Applied Biosystems). Amplification and detection were performed using the ViiA 7 platform (Applied Biosystems). Thermal cycling conditions were 10 min at 95 °C followed by 50 two-step cycles, including 15 s of denaturation at 92 °C and 60 s of annealing/extension at 60 °C. All reactions were performed in duplicate and allele calling was done using QuantiStudio software; only genotypes with an automatic call rate >95% were considered, error rate was <3%. Allele calling was double-checked manually in the raw data plot, comparing the amplitude and kinetics of fluoresce patterns. Samples that failed to provide a genotype were repeated in additional reactions. All genotyping experiments, including DNA isolation, DNA quantification/quality control, and genotyping were performed at the OSTEOimmunology laboratory, Bauru School of Dentistry University of Sao Paulo (FOB/USP).

### 2.3. DNA-DNA Checkerboard

Subgingival biofilm samples were collected from nine subgingival sites of a fraction of our total sample (*n* = 146; 69 cases and 77 controls), and were assayed for the presence and quantity of 40 bacterial species [11,56] (Table 3), as extensively described elsewhere [11,57,58]. In brief, after periodontal examination three deep (>5 mm), three medium (4–5 mm) and three shallow (1–3 mm) periodontal sites were selected for microbiological sampling. After relative isolation with gauze and cotton rolls, a sterile Gracey curette (Hu-Friedy, Chicago, IL, USA) was gently introduced into the bottom of the periodontal pocket and then rinsed in a tube containing 150 µL of TE buffer, then 100 µL NaOH 0.5 M were added and the sample was agitated for 1 min. The nine samples per subject were pooled together. Later, the samples were boiled for 10 min and 800 µL of fresh 5 M NH_4_ acetate were added. One milliliter of the mixture was placed in each individual lane on a nylon membrane using a Minislot device (Immunetics, Cambridge, MA, USA). After fixation of the DNA to the membrane, the membrane was placed in a Miniblotter 45 (Immunetics), with the lanes of DNA at a 90-degree angle with respect to the lanes of the device. Digoxigenin-labeled whole-genome DNA probes for 40 subgingival species were hybridized in individual lanes of the Miniblotter 45. After hybridization, the membranes were washed at high stringency and the DNA probes were detected using a digoxigenin-specific antibody conjugated with alkaline phosphatase. Signals were detected using AttoPhos substrate (Amersham Life Sciences, Arlington Heights, IL, USA), and results were read using a Typhoon Trio Plus variable mode imager (Molecular Dynamics, Sunnyvale, CA, USA). Two lanes in each run contained standards with 10^5^ or 10^6^ cells of each species. Signals evaluated using the Typhoon Trio Plus variable mode imager were converted to absolute counts by comparison with the standards on the same membrane. Failure to detect a signal was recorded as zero.

### 2.4. Statistical Analysis

Compliance with the Hardy-Weinberg equilibrium for each SNP was tested by a chi-square test. Standard and allelic case/control association analysis with disease phenotype were performed using a chi-square test and Fisher’s exact test. Significant associations between phenotype/genotype and bacterial counts were established by the two-stage step-up adaptive method [59]. Briefly, in the first stage, the genotype (represented as a binary variable by allele-carrying) was sequentially tested against the counts for each bacterial species, obtaining a series of *p*-values. The distribution of these *p*-values was used to estimate the fraction of null hypothesis that were actually true. In the second stage, a reductive iterative process determined which *p*-values were low enough to be considered discoveries. A Q value of 5% was accepted as the maximum false positive rate. This adaptive procedure greatly diminishes the probability of false positives in repetitive testing.

A *p*-value < 0.05 was considered significant. Analyses were performed in GraphPad Prism v7.02 (GraphPad software, La Jolla, CA, USA), Stata14 (Stata Corp, College Station, TX, USA) and PLINK v1.07 [60].

## 3. Results

SNPs rs7762544 and rs11695297 failed the Hardy-Weinberg equilibrium test and were excluded from subsequent analysis (Table 4). There were no mutant homozygous subjects in the control group of rs3826782, thus it was excluded from the case/control association analysis (Table 4).

The case/control association test did not demonstrate a significant effect of any of the 16 assessed SNPs with the disease phenotype (Table 4). No further association analysis with disease phenotype were performed.

**Figure 1:**
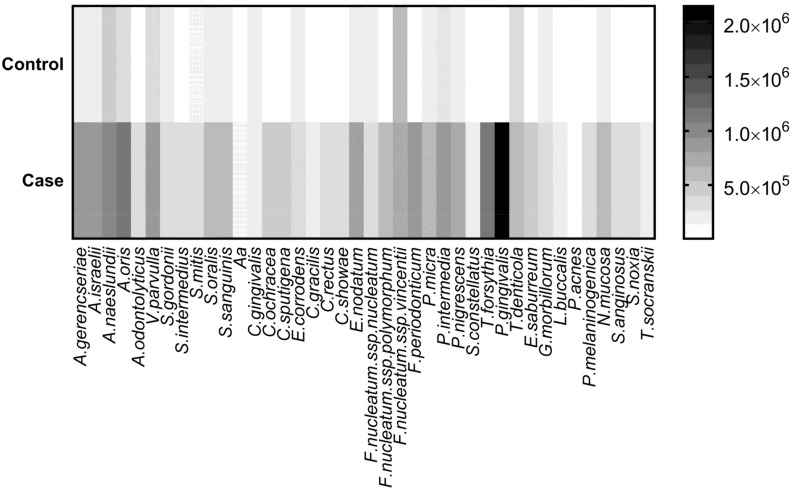
Heat map of bacterial counts for 40 subgingival species belonging to the classic subgingival microbial complexes in controls (*n* = 77) and cases (chronic periodontitis) (*n* = 69).

The two-stage linear set-up procedure [59] discovered 36 significantly different bacterial counts between control and CP individuals (Figure 1 and Table 5).

**Figure 2:**
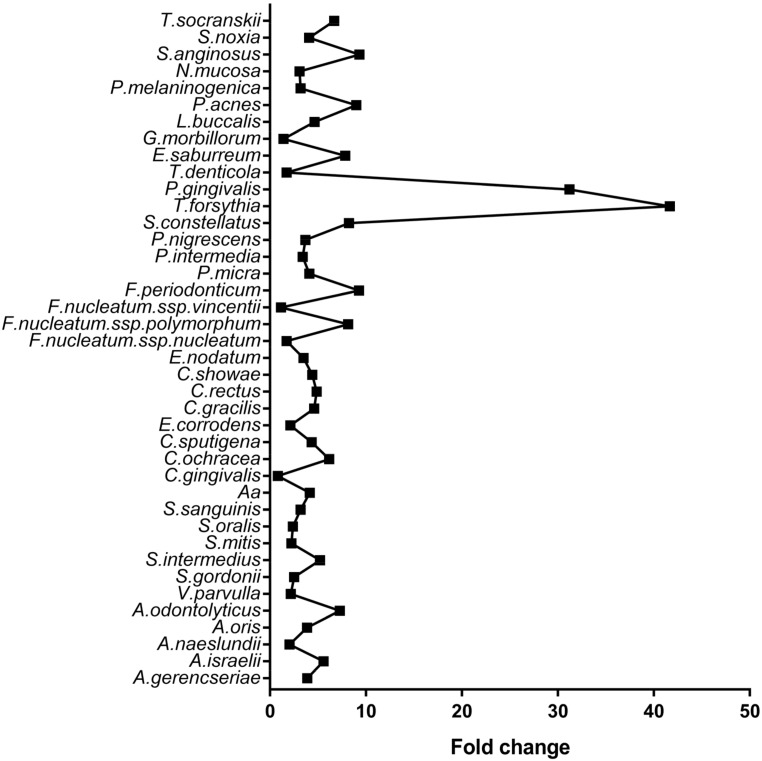
Fold change of bacterial counts in cases versus control subjects for 40 subgingival species belonging to the classic subgingival microbial complexes in controls (*n* = 77) and cases (chronic periodontitis) (*n* = 69).

Bacterial counts increased significantly for almost all tested species in cases versus controls, with *Treponema denticola* and *Porphyromonas gingivalis* demonstrating a 41 and 31-fold increase, respectively (Figure 2).

SNPs in Hardy-Weinberg equilibrium (*n* = 17) were tested for discovery of significant changes in subgingival microbiological pattern in the chronic periodontitis group.

**Figure 3:**
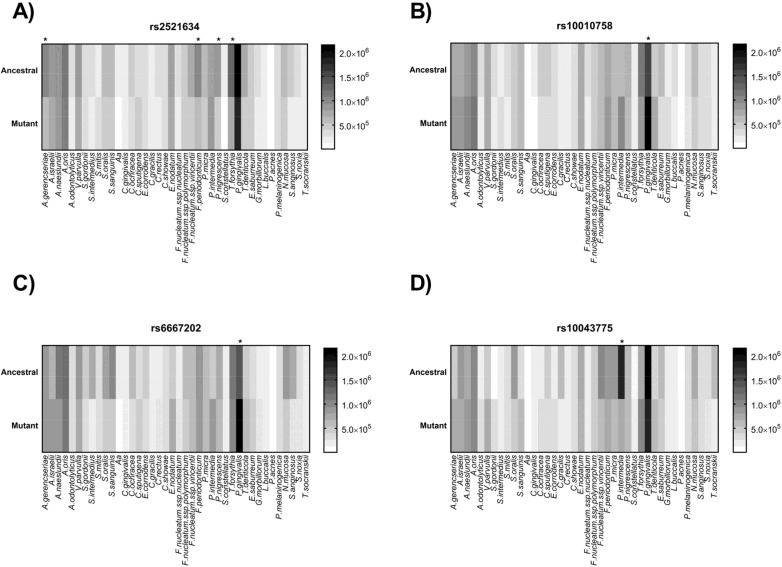
Heat maps of bacterial counts for 40 subgingival species in ancestral and mutant allele-carrying chronic periodontitis subjects. (**A**) rs2521634. (**B**) 10010758. (**C**) rs6667202. (**D**) rs10043775. *: discovery by two-stage linear set-up procedure Benjamini, Krieger, and Yekutieli (Q = 5%).

The mutant allele for the polymorphisms rs2521634 proved significantly associated with decreased counts of *Tannerella forsythia*, *Actinomyces gerencseriae*, *Fusobacterium periodonticum*, and *Prevotella nigrescens* (Figure 3A). Discoveries were *T. forsythia* *p*-value = 0.001; *A. gerencseriae* *p*-value = 0.02; *F. periodonticum* *p*-value = 0.03; and *P. nigrescens* *p*-value = 0.03.

The mutant allele for the polymorphism rs10010758 proved significantly associated with increased counts of *P. gingivalis* (Figure 3B). Discovery was *P. gingivalis* *p*-value = 0.01.

The mutant allele for the polymorphism rs6667202 proved significantly associated with increased counts of *P. gingivalis* (Figure 3C). Discovery was *P. gingivalis* *p*-value = 0.005.

The mutant allele for the polymorphism rs10043775 proved significantly associated with decreased counts of *Prevotella intermedia* (Figure 3D). Discovery was *P. intermedia* *p*-value = 0.02.

The remaining 13 SNPs failed to pass the discovery threshold for *p*-value adjustment of the two-stage linear set-up procedure and were considered not associated with changes in the subgingival microbiological pattern (data not shown).

## 4. Discussion

Periodontitis is a complex disease, triggered by the presence of dysbiotic microbial communities, modulated by genetic factors, and modified by the host’s immune response, which can be either protective or destructive [7]. Genetic variants determine at least 50% of the differential susceptibility profiles of periodontitis [20]. Therefore, it is possible to hypothesize that genetic variations that facilitate colonization of subgingival biofilm by keystone microbes and/or facilitate the establishment of dysbiotic subgingival communities could act as primary risk factors for the development of periodontitis, since this initial *step* would subsequently lead to unbalance host-microbe homeostasis and afterward to tissue breakdown [13,61,62]. Indeed, the SNPs selected for this study had been previously associated with the occurrence of periodontitis or with changes on the subgingival biofilm in GWAS [27,30]. While a recent systematic review states that definitive links between genetic polymorphisms association with subgingival microbiota are still required [43], increasing evidence points to correlations between SNPs and periodontopathogens’ frequency of detection and load [39,40,41,42].

Initially, from the classic case/control genetic association viewpoint, the association test failed to demonstrate any significant effect of the tested SNPs on periodontitis risk. This is somewhat expected, since our sample is underpowered to detect the small genetic effects over the disease phenotype that these SNPs probably exert [63]. In fact, our study was primarily intended to test the interaction between the selected SNPs and the subgingival microbiological pattern. Testing for association for the disease phenotype would require a larger sample, in which it would be impractical to perform such a comprehensive microbiological profiling as the one carried out in the present study. Indeed, previous evidence has demonstrated that several SNPs exert significant modulatory effects on inflammatory biomarkers but do not always provoke a differential risk phenotype [24,35,37,38]. Yet, qualitative changes in the subgingival biofilm can overcome the genetic predisposition to increased expression of inflammatory biomarkers, independently modulating the response [36,37,38]. From this perspective, the direct association of SNPs and changes in the subgingival microbiota appear as important outcomes, since the genetic profile provides the context in which the biofilm develops.

Notwithstanding the controversies and limitations regarding the genetic case/control association studies, our results are in line with the published evidence, since four of the tested SNPs, namely rs2521634, rs10010758, rs6667202 and rs10043775, proved significantly associated with changes in the subgingival microbiological pattern. Notably, the strategy used to characterize the subgingival biofilm in our study was more broad and sensitive than the strategy used in the cited GWAS [27,30]. We tested for 40 subgingival species, including all species belonging to the classic subgingival microbial complexes [11]. This approach allows us to gain an insight to the effect of the polymorphism over the subgingival microbiological pattern as a whole. Metagenomic data has proven that changes in the diversity of subgingival biofilm could be correlated with disease status and predict progressive sites [64]. Indeed, the strategy used to unveil the possible effect of the mutations in the subgingival microbiological pattern in the chronic periodontitis sample, coupled with a statistical analysis designed to adaptively adjust for false positives [59,65], proved powerful enough to detect significant microbiological changes associated with the polymorphic variations. Even though the unit of observation for the statistical analysis remained the subject, it is important to bear in mind that each subject was genotyped for 19 SNPs and that the microbiological profiling was the result of sampling 9 different subgingival sites per subject, with each one tested independently for 40 species. We believe that the amount of independent data included in each analysis strengthened our results, even in a relatively small sample [66]. Also, we purposefully excluded smokers (including former smokers) and patients presenting metabolic diseases know to modify periodontitis phenotype. These major disease co-factors have the potential to obscure the phenotypic characterization, dampening the power of subsequent analysis [67,68].

Regarding the SNPs associated with periodontal microbes, SNP rs2521634, located near the NPY gene, has been previously associated with the occurrence of severe chronic periodontitis [27,30]. Our results demonstrated that mutant allele-carriers were at decreased risk of harboring *T. forsythia*, *A. gerencseriae*, *F. periodonticum*, and *P. nigrescens*, regarded as disease-associated bacteria [11,58]. This result is coherent with GWAS results that demonstrated a pooled estimate effect for severe chronic periodontitis of odds ratio (OR) = 1.49, 95%, confidence interval (CI) = 1.28–1.73, P = 3.5 × 10^−7^ for the ancestral allele (G). Therefore, the mutant allele for rs2521634 (A) is protective for severe chronic periodontitis, and arguably mutant allele-carrier subjects would be at decreased risk of harboring periodontal pathogens. Although it is impossible to trace a complete parallel between our results and the GWAS results, the fact that the direction of the association is the same increases our confidence in the reality of the association. It is noteworthy that our Brazilian subjects and the European population tested on the cited GWAS have very different genetic backgrounds [55,69]. The fact that the association was replicated in a Brazilian cohort (characteristically described as a mosaic genome from Amerindian, European and African ancestries) [53,54,55], supports the notion of an important role of this polymorphism conferring differential susceptibility for periodontitis. In terms of mechanistically linking neuropeptide Y (*NPY*) to periodontal microbial patterns, the literature in this field is particularly scarce. A previous study demonstrated that NPY did not have direct antimicrobial activity against oral microorganisms, namely *S. mutans*, *C. albicans*, and *A. actinomycetemcomitans*, but it suggests that a stimulatory action over local epithelial cells to produce other innate immune factors like defensins and cathelicidin could account for antimicrobial effects [70]. Additional evidence that NPY could be involved in the maintenance of host-microbe homeostasis at the periodontium derives from the observation that NPY levels are higher in human gingival crevicular fluid in healthy conditions when compared to periodontitis [71]. Additionally, recent evidence has demonstrated immune modulatory functions for NPY, inhibiting the recruitment of monocytes in severe infections of the central nervous system [72]. Similarly, in vivo experiments suggest that NPY agonists are effective in diminishing the blood title of TNFα in endotoxin-induced septic shock [73]. Hypothetically, it is plausible to argue that NPY has a regulatory effect in the response against subgingival microbes, and that the mutant-allele for rs2521634 confers protection against periodontal pathogens by a mechanism associated with the infiltration of monocytes and TNFα secretion in periodontal tissues.

Our results also demonstrated an association of the mutant allele (C) for rs10010758, located in an intronic region of the TBC1 domain family member 1 (*TBC1D1*) gene, with significantly increased counts of the *red complex* pathogen *P. gingivalis*. This result is in line with previous evidence demonstrating that SNP rs10010758 is associated with increased risk of harboring ‘red complex’ disease-associated bacteria (OR = 1.91, 95% CI = 1.45–2.51, P = 3.7 × 10^−6^) for the mutant allele [27]. Again, the fact that the result was replicated with identical direction of association is suggestive of a real effect of the polymorphism over the composition of the subgingival microbiota. Further, the effect seems to be conserved in populations with different ethnic backgrounds. The possible mechanism of action of this association is uncertain since there is no a single piece of evidence of a possible link between TBCD1 (Rab GTPase activating protein) and immune functions. However, recent studies in mice can point towards indirect mechanistic links between TBCD1 and periodontitis. It has been demonstrated that the deletion of TBC1D1 modifies glucose, lipid, and energy homeostasis impacting insulin resistance, body fat metabolism, leading to the development of obesity [74,75]. Interestingly, recent studies also point to an association between subgingival bacterial counts, inflammation, and insulin resistance [76,77]. In addition, obesity has been associated with increased levels and proportions of periodontal pathogens and specifically high prevalence of *P. gingivalis* [78,79]. Consequently, there are two discrete possibilities to explain the association with changes in the subgingival microbiota. First, the mutation may be involved in conferring differential expression or functional properties to TBCD1, which may be linked to changes in the host/pathogen barrier by a direct mechanism that has not yet been described or by an indirect mechanism linked to insulin resistance and fat metabolism. Furthermore, the mutation could be in linkage disequilibrium with another mutation that is truly responsible for the changes in the subgingival microbiota [31]. At this point, it is mandatory to consider that the lack of studies focused on TBCD1 functions make the prior possibilities highly speculative and further studies are required to provide a more solid mechanistic link between genetic variation of TBCD1 and periodontitis or its microbiological surrogates.

Additionally, SNP rs6667202, located near the interleukin 10 (*IL10*) gene, mutant allele-carriers (A) demonstrated a significant increase in the counts of *P. gingivalis*. This result is concordant with previous evidence associating ancestral allele-carriers (C) for rs6667202 with decreased risk of aggressive periodontitis (OR = 0.77, 95% CI = 0.6–0.95, P = 0.016) in a German/Austrian population [28]. IL10 is a key regulatory cytokine involved in the suppression of inflammation and return to homeostatic state [80] and extensive evidence links increased levels of IL10 with resistance to inflammatory bone loss in experimental periodontitis [81,82]. However, in addition to actively suppressing inflammatory mechanisms, IL10 can interfere in some antimicrobial responses, such as T_h_1-type responses that are involved in the control of periodontopathogens [83,84]. A recent study demonstrated that IL10 genetic deficiency leads to significant taxonomic changes in the gut microbiome [85], suggesting that a similar effect may take place in the periodontal environment. Therefore, the existence of differential patterns of subgingival infection in association with distinct IL10 genotypes appears biologically plausible and may contribute to the development of periodontitis, allowing the establishment of a dysbiotic subgingival microflora.

Likewise, for SNP rs10043775 missense variant of the TBXO38 gene which encodes the F-box protein 38 that contains an F-box domain and may participate in protein ubiquitination by E3 ubiquitin ligase complex, but whose exact functions remains unclear, [86] our data demonstrate that ancestral allele-carriers exhibited a 3-fold increase in the counts of *P. intermedia*, which is an ‘orange complex’ bacteria and regarded as a periodontal pathogen [11,87,88]. F-box proteins might function as transcription factors, *FBXO38* being particularly associated with the KLF transcription factors family, widely expressed in the developing nervous system [86]. Our results are in concordance with previous reports associating a Han Chinese population of ancestral allele-carriers with increased risk of suffering severe chronic periodontitis (OR = 1.24, P = 0.0009) [29]. The possible mechanism of association between rs10043775 and changes in the subgingival microbiological pattern is completely uncertain, since there is no direct evidence of *FBXO38* association with host response. However, due its putative role in the ubiquitination process, which in turn has been implicated in the immune response, it could exert some impact over periodontal microbiota via this pathway [89]. However, it is possible that a yet unknown mechanism is responsible for the differential susceptibility to periodontitis demonstrated by our sample, or alternatively that rs10043775 is in linkage disequilibrium with another variant that is truly responsible for the changes in the subgingival microbiological pattern. Thus, further studies are required to provide a solid link between this genetic variation and the microbiological changes in periodontal tissues leading to periodontitis.

Despite the lack of extensive studies in the field to support a solid and integrative mechanistic association between the genetic variations and the periodontal microbiota, it is necessary to highlight that the Brazilian cohort investigated in this study, the European population used in three other studies [27,28,30], and the Han Chinese population used in one study [29] possess very distinct genetic backgrounds [55,69,90]. The fact that the associations were maintained despite the genetic distinction between such populations is indicative of an important and conserved role for these four polymorphisms in the host/microbe barrier. It is necessary to clarify that the classic ancestry stratification based in phenotypic features was not performed in this study due to the high ancestral variability observed in Brazilian population, considered a singular trihybrid (European, African, and Amerindian) ancestry, which is weakly represented by phenotypic characteristics [53,54,55]. Furthermore, since both cases and controls were recruited in the same geographical region and taking into consideration that none of the criteria used in the recruitment process resulted in stratified sampling, we remain confident that no ethnic sampling bias was incorporated in the recruited population. Certainly, the mosaic nature characteristically observed in Brazilian populations’ genotypes [53,54,55] suggests that genetic associations derived from such complex genetic backgrounds may be relevant in a broader and diverse population context, in contrast with findings derived from populations with a narrower genetic variation.

From an evolutionary perspective, host-pathogen interactions have been considered important signals of natural selections of modern humans to local conditions [91]. Indeed, infectious pathogens are arguably among the strongest selective forces that act on human populations [92]. Pathogens drive selection on genetic variants that affect resistance and include pathogens that cause acute illnesses or chronic infections, either through death or poor health, and impair nutrition, growth, cognitive development, and fertility [92]. While periodontitis’ direct effects are limited to the local tooth-supporting tissues, it may indirectly impact several systemic conditions [93], and its potential involvement in evolutionary natural selection remains unexplored. Still, since periodontitis is unlikely to cause direct natural selection or alter the population mating structure, it is necessary to consider that the selective pressures shaping the response against periodontal pathogens are possibly the expression of adaptations triggered by non-oral conditions. In this setting, it is possible to hypothesize that the adaptive bias to T helper 2 cell/ regulatory T cell (T_h_2/Treg) responses to anticipate helminth colonization and the immune deregulation resulting from the ‘hygienic’ life conditions of modern humans are important forces shaping the phenotypic presentation of periodontitis [94]. The fact that two (NPY and IL10) out of four SNPs associated with changes in the subgingival microbial pattern are part of immune modulatory pathways seems to support this theory. Indeed, IL10 promoter has been described as an important element in balancing selection, resulting in significant phenotypic effects. Increased IL10 secretion might be advantageous in some environmental scenarios but not in others and consequently contribute to drive evolutionary change [95].

Nonetheless, at this point any evolutionary interpretation is highly speculative in view of the lack of additional data in the literature in this field to support deeper discussion. However, from the clinical perspective, the association between genetic variants and periodontitis-associated microbiota presents an interesting framework of host-pathogen interaction. The periodontal disease process involves multiple mechanisms that lead to tissue destruction (which ultimately serves as basis for the clinical parameter measurements), and consequently, the direct association between clinical parameters values and genetic variants presents an inherent complexity that may limit the strength of such data. Indeed, even studies that positively associate SNPs with periodontitis risk may fail in providing direct association between the genetic variants and specific clinical readouts [34,35,36,37,38]. The association between specific SNPs with red- and orange-complex pathogenic bacteria (associated with chronic periodontitis development) [57,58,96] may provide a more direct link towards the identification of ‘periodontitis susceptible’ genotypes, which may impact the clinic management of this condition.

In conclusion, we presented strong evidence supporting a direct connection between the host’s genetic profile, specifically rs2521634, rs10010758, rs6667202, and rs10043775 polymorphisms and the occurrence of chronic periodontitis-associated bacteria. The pathway explored in this study must be expanded to include more bacterial species and more SNPs identified by unbiased methods, in order to construct a clearer picture of the complex relationship established at the periodontal host/pathogen barrier.

## Figures and Tables

**Table 1:** Clinical and demographical data of the recruited cases and healthy controls.

	Healthy (*n* = 91)	Chronic Periodontitis (*n* = 76)
*Gender distribution*	*50 f/41 m*	*39 f/37 m*
Age	45.1 ± 5.9	46.8 ± 6.1
*Clinical parameters*	*value* ± *SD*	*value* ± *SD*
Probing depth	2.2 ± 0.6	4.5 ± 0.7
Clinical Attachment Loss	0.6 ± 0.2	4.2 ± 0.7
% Bleeding on probing	4.5 ± 2.7	66.2 ± 8.9
Plaque index	30.2 ± 6.2	56.9 ± 9.1

**Table 2:** Nineteen single-nucleotide polymorphisms (SNPs) assayed in the sample population. Association refers to previously published evidence linking the polymorphism with an outcome or phenotype. MAF *: minor allele frequency, latest release of phase 2 1000 genome project; NCTEV: non-coding transcript exon variant; CP: chronic periodontitis; AP: aggressive periodontitis; Aa: *Aggregatibacter actinomycetemcomitans*.

Code	Nearby Gene	Allele	Ancestral	Chrm.	Position	Association	MAF *	Relative Position
rs2521634	*NPY*	G/A	G	7	37614913	Severe CP	0.24	Intergenic variant
rs7762544	*NCR2*	G/A	G	6	147785313	Severe Chronic Periodontitis	0.15	Intergenic variant
rs12032672	*PKN2*	A/C	A	1	74883781	Red complex	0.44	Intergenic variant
rs10010758	*TBC1D1*	T/C	T	4	1842012	Red complex	0.29	Intron variant
rs1932040	*RUNX2*	A/G	A	6	88398224	Orange complex	0.4	Intergenic variant
rs9942773	*CSMD3*	A/C	C	8	22115347	Orange complex	0.19	Intergenic variant
rs1616122	*VAMP3*	C/T	C	1	137669543	Orange complex	0.42	Intron variant
rs11621969	*FOS*	T/C	T	14	7444172	Aa	0.15	Intergenic variant
rs9287989	*WAPAL*	T/C	C	2	45804766	Aa	0.5	Regulatory region variant
rs8080364	*KIAA0753*	C/T	T	17	24344565	Control	0.27	NCTEV
rs2836981	*BRWD1-IT2*	G/A	G	21	39610235	Control	0.39	Intron variant
rs11695297	*MYT1L*	T/A	A	2	22088619	Control	0.47	Intron variant
rs1537415	*GTL6D1*	G/C	G	9	6838736	AP	0.31	Intron variant
rs1333048	*CDKN2A/2B*	A/C	A	9	43163827	AP; CP	0.45	Downstream gene variant
rs6667202	*IL10*	C/A	C	1	205023715	AP	0.31	Intergenic variant
rs3826782	*VAV1*	G/A	G	19	41487293	Severe Chronic Periodontitis	0.15	Intron variant
rs10043775	*FBXO38*	T/C	C	5	6478800	Red complex	0.26	Missense variant
rs4794067	*TBX21*	T/C	C	17	176425987	Chronic Periodontitis	0.25	Upstream gene variant
rs2891168	*CDKN2B-AS1*	A/G	A	9	115190203	Aggressive Periodontitis	0.42	Intron variant

**Table 3:** Bacterial species assayed by DNA-DNA hybridization checkerboard.

Bacterial Species Assayed by DNA-DNA Hybridization Checkerboard			
*Actinomyces gerencseriae*	*Streptococcus sanguinis*	*Fusobacterium nucleatum* spp. *nucleatum*	*Treponema denticola*
*Actinomyces israelii*	*Aggregatibacter actinomycetemcomitans*	*Fusobacterium nucleatum* spp. *polymorphum*	*Eubacterium saburreum*
*Actinomyces naeslundii*	*Capnocytophaga gingivalis*	*Fusobacterium nucleatum* spp. *vincentii*	*Gemella morbillorum*
*Actinomyces oris*	*Capnocytophaga ochracea*	*Fusobacterium periodonticum*	*Leptotrichia buccalis*
*Actinomyces odontolyticus*	*Capnocytophaga sputigena*	*Parvimonas micra*	*Propionibacterium acnes*
*Veillonella parvula*	*Eikenella corrodens*	*Prevotella intermedia*	*Prevotella melaninogenica*
*Streptococcus gordonii*	*Campylobacter gracilis*	*Prevotella nigrescens*	*Neisseria mucosa*
*Streptococcus intermedius*	*Campylobacter rectus*	*Streptococcus constellatus*	*Streptococcus anginosus*
*Streptococcus mitis*	*Campylobacter showae*	*Tannerella forsythia*	*Selenomonas noxia*
*Streptococcus oralis*	*Eubacterium nodatum*	*Porphyromonas gingivalis*	*Treponema socranskii*

**Table 4:** Genotype count and frequencies for all tested SNPs. HZ: homozygous; AF: allele frequency; H-W: Hardy-Weinberg equilibrium chi-square test; Chi-square/*p*-value: case/control association test with disease phenotype; NA: non-available.

SNP	Control [n (%)]				CP [n (%)]				Data Analysis		
	Ancestral HZ	HetZ	Mutant HZ	Mutant AF	Ancestral HZ	HetZ	Mutant HZ	Mutant AF	H-W	Chi-Square	*p*-Value
rs2521634	46 (51.7)	31 (34.8)	12 (13.4)	36 (25)	39 (54.1)	30 (41.6)	3 (4.1)	55 (30.8)	0.443	4.245	0.12
rs7762544	7 (9.6)	20 (27.3)	46 (63)	112 (76.7)	0 (0)	22 (33.3)	44 (66.6)	110 (83.3)	0.048	NA	NA
rs12032672	25 (40.3)	30 (48.3)	7 (11.3)	44 (35.5)	20 (31.2)	35 (54.7)	9 (14.1)	53 (41.4)	0.65	1.159	0.56
rs10010758	22 (62.8)	11 (31.4)	2 (5.7)	15 (21.4)	32 (57.1)	16 (28.6)	8 (14.3)	32 (28.5)	0.33	1.168	0.44
rs1932040	3 (3.6)	33 (39.3)	48 (57.1)	129 (76.7)	6 (8.2)	36 (49.3)	31 (42.5)	98 (67.1)	0.44	4.038	0.13
rs9942773	4 (5)	27 (33.7)	49 (61.2)	125 (78.1)	6 (9.4)	19 (29.7)	39 (60.9)	97 (75.7)	0.6	1.164	0.55
rs1616122	27 (31.8)	35 (41.1)	23 (27)	81 (47.6)	14 (18.4)	43 (56.6)	19 (25)	81 (53.2)	0.057	4.835	0.08
rs11621969	60 (71.4)	22 (26.2)	2 (2.4)	26 (15.4)	49 (64.5)	24 (31.5)	3 (3.9)	30 (19.7)	0.75	0.999	0.61
rs9287989	28 (32.5)	47 (54.6)	11 (12.7)	69 (40)	22 (29.7)	37 (50)	15 (20.2)	67 (45.2)	0.111	1.635	0.44
rs8080364	37 (44)	39 (46.4)	8 (9.5)	55 (32.7)	46 (60.5)	27 (35.5)	3 (3.9)	33 (21.7)	0.282	5.043	0.08
rs2836981	14 (16.1)	42 (48.3)	31 (35.6)	104 (59.7)	7 (9.2)	40 (52.6)	29 (38.1)	98 (64.4)	0.808	1.714	0.42
rs11695297	43 (53.7)	25 (31.2)	12 (15)	49 (30.6)	20 (27.4)	38 (52)	15 (20.5)	68 (46.5)	0.029	NA	NA
rs1537415	12 (14.1)	37 (43.5)	36 (42.3)	109 (64.1)	6 (7.9)	39 (51.3)	31 (40.8)	101 (66.4)	0.97	1.929	0.38
rs1333048	17 (25)	33 (48.5)	18 (26.4)	69 (50.7)	16 (25.8)	28 (45.1)	18 (29)	64 (51.6)	0.993	0.1636	0.92
rs6667202	12 (13.6)	30 (34.1)	46 (52.2)	122 (69.3)	10 (13.1)	35 (46)	31 (40.8)	97 (63.8)	0.191	3.365	0.18
rs3826782	64 (78)	18 (21.9)	0 (0)	18 (10.9)	57 (76)	16 (21.3)	2 (2.6)	20 (13.3)	0.204	NA	NA
rs10043775	5 (6)	24 (28.9)	54 (65.1)	132 (79.5)	4 (5.4)	26 (35.1)	44 (59.4)	114 (77)	0.57	1.159	0.56
rs4794067	8 (9.3)	31 (36)	47 (54.6)	125 (72.6)	3 (3.9)	28 (36.8)	45 (59.2)	118 (77.6)	0.367	3.216	0.2
rs2891168	20 (29.8)	31 (46.2)	16 (23.9)	63 (47)	18 (28.6)	33 (52.3)	12 (19)	57 (45.2)	0.998	0.761	0.68

**Table 5:** Significantly altered bacterial counts of subgingival microorganisms between control and CP subjects. Adjusted *p*-value: Discovery by two-stage linear set-up procedure Benjamini, Krieger, and Yekutieli (Q = 5%), sorted from the lowest to highest significant *p*-value.

Microorganism	Control	CP	Adjusted *p*-Value
*A. israelii*	160,345	893,511	<0.001
*P. gingivalis*	68,876	2,150,036	<0.001
*A. oris*	302,836	1,171,655	<0.001
*T. forsythia*	26,506	1,104,569	<0.001
*F. periodonticum*	98,231	910,910	<0.001
*A. gerencseriae*	235,808	913,498	<0.001
*P. intermedia*	259,762	882,319	<0.001
*E. nodatum*	228,546	795,966	<0.001
*P. nigrescens*	188,205	692,758	<0.001
*P. micra*	161,019	659,696	<0.001
*A. naeslundii*	468,479	957,118	<0.001
*F. nucleatum* spp. *polymorphum*	67,973	552,734	<0.001
*V. parvulla*	385,381	837,456	<0.001
*E. saburreum*	61,402	480,620	<0.001
*N. mucosa*	200,501	615,542	<0.001
*S. sanguinis*	189,625	603,031	<0.001
*C. ochracea*	68,674	423,281	<0.001
*C. sputigena*	99,803	433,313	<0.001
*S. anginosus*	39,551	368,112	<0.001
*S. oralis*	232,754	552,347	<0.001
*T. denticola*	374,527	648,599	<0.001
*A. odontolyticus*	42,836	311,538	<0.001
*C. showae*	73,988	325,362	<0.001
*P. melaninogenica*	111,172	353,281	0.001
*S. noxia*	75,718	308,615	0.001
*S. intermedius*	54,897	285,286	0.001
*C. rectus*	59,601	289,257	0.001
*S. gordonii*	146,660	368,106	0.001
*E. corrodens*	183,801	388,124	0.002
*T.socranskii*	33,894	226,699	0.003
*L. buccalis*	52,565	244,344	0.003
*C. gracilis*	48,808	224,658	0.005
*S. mitis*	137,170	306,687	0.006
*S. constellatus*	23,077	190,087	0.007
*F.nucleatum* spp. *nucleatum*	162,832	280,366	0.025
*A. actinomycetemcomitans*	32,715	135,463	0.034
